# Respiratory Syncytial Virus Epidemiology During and After Covid‐19 Pandemic in Africa: Systematic Review and Meta‐Analysis

**DOI:** 10.1002/hsr2.71583

**Published:** 2025-11-26

**Authors:** Moussa Issa, Adamou Lagare, Goni A. M. Bachir, Arnol Bowo ‐Ngandji, Fatimata Hassane, Larwanou Harouna Magagi, Doutchi Mahamadou, Haoua Seini, Eric Adehossi, Alzouma Mayaki Zoubeirou

**Affiliations:** ^1^ Centre de Recherche Médicale et Sanitaire (CERMES) Niamey Niger; ^2^ Département de Biologie, Faculté des Sciences et Techniques Université Abdou Moumouni de Niamey, BP Niamey Niger; ^3^ Université André Salifou Zinder Niger; ^4^ Département de Microbiologie Université Yaoundé Cameroon; ^5^ Hôpital Général de Référence Niger

**Keywords:** Africa, covid‐19, prevalence, respiratory syncytial virus

## Abstract

**Background and Aims:**

Respiratory syncytial virus (RSV) is a major agent of acute respiratory infections in children and the elderly. RSV epidemiology has been changed by the since Covid‐19 pandemic and this review aimed to assess the extent of this change in Africa.

**Methods:**

We searched Medline, Embase, Global Health, Web of Science, and Africa Index Medicus for studies reporting RSV epidemiology during and after the pandemic. We assessed heterogeneity using the *I*² statistic and evaluated study quality with the Hoy et al. checklist for prevalence studies. Publication bias was assessed with the Egger test. Pooled estimates of prevalence and incidence were calculated using a random‐effects model. Analyses were stratified by pandemic era.

**Results:**

Nineteen studies from 12 African countries, including 53,550 patients, met the inclusion criteria. The pooled prevalence of RSV infection was 13.0% (95% CI 9.5–17.1), with substantial heterogeneity (*I*² = 99.2% [99.1–99.3]). The Egger test showed no evidence of publication bias (*p* = 0.745). Prevalence was highest in children (29.8% [18.8–42.1]) compared with all‐age populations (5.9%; *p* < 0.001), and in hospitalized patients compared with outpatients (21.3% vs. 11.3%; *p* < 0.001). In the post‐pandemic period, prevalence rose significantly to 30.6% (12.4–52.5), compared with 8.8% (3.7–15.7) during the pandemic (*p* = 0.071). The overall incidence of RSV infection was 3.0 per 1000 (1.8–4.2) person‐year.

**Conclusion:**

This systematic review highlights a marked resurgence of RSV in Africa following the easing of COVID‐19 restrictions, particularly among children. These findings underscore the urgent need for strengthened RSV surveillance, targeted prevention strategies, and expanded access to new vaccines and monoclonal antibodies.

## 1 Introduction

Respiratory syncytial virus (RSV) remains a leading cause of acute lower respiratory infections (ALRI), particularly in young children, older adults, immunocompromised individuals, and those with chronic comorbidities. These populations are vulnerable to severe disease and often require hospitalization or intensive care [1, 2]. Globally, RSV is associated with approximately 33.1 million episodes of ALRI each year in children under five, leading to an estimated 3.6 million hospitalizations and 23,600 deaths; most of which occur in low‐income countries [3, 4]. In temperate countries, RSV epidemics follow a seasonal pattern with peaks during winter months, especially between December and January [5]. In contrast, tropical and subtropical countries often report endemic RSV circulation throughout the year, with increased incidence, during the rainy seasons [6]. The virus is highly contagious and spreads primarily through respiratory droplets and contact with contaminated surfaces. Reinfection is common throughout life due to limited long‐term immunity, and RSV continues to pose a recurrent burden on health systems worldwide. RSV has two antigenic subgroups, RSV‐A and RSV‐B, which are defined based on variability in the G glycoprotein gene [7]. Some studies suggest that infections caused by RSV‐A may lead to more severe disease [8]. In clinical settings, common symptoms include cough, nasal congestion, rhinorrhoea, feeding difficulties, and respiratory distress. RSV is also a major contributor to nosocomial infections, particularly in pediatric wards, where it can spread via healthcare workers or fomites [9, 10].

While the recent advances in vaccines and monoclonal antibody development offer hope for prevention, disparities in access to these tools persist [11]. Palivizumab remains the used prophylactic option, but its high cost limits accessibility in many low‐income settings. New long‐acting monoclonal antibodies have recently been approved in the US and Europe and could improve infant protection [12, 13]. Ribavirin, the only antiviral, is rarely used due to limited efficacy and high toxicity [14].

The Covid‐19 pandemic disrupted the transmission dynamics of many respiratory viruses, including RSV. Public health interventions, such as lockdowns like mask use, social distancing and improved hand hygiene led to a sharp decline in RSV circulation globally during 2020—several large‐scale studies have documented this effect [15, 16]. In a global meta‐analysis by Cong et al., hospitalization rates for RSV‐associated ALRI in children under five fell sharply in 2020 across high‐income, upper‐middle‐income, and middle‐income settings [17]. However, by 2022, a strong resurgence was observed in high‐income countries, while hospitalization rates remained below pre‐pandemic levels in middle‐income regions such as Kenya. The study found a shift in the age distribution of hospitalized children, with higher proportions of RSV cases in older infants and toddlers, likely due to immunity gaps created by missed seasonal exposures during the pandemic. Another meta‐analysis by Zhang et al. evaluated RSV prevalence in China during pre‐pandemic, lockdown, and post‐lockdown periods. The authors found a significant drop in RSV‐related hospitalizations during lockdown, followed by a sharp increase post‐lockdown as restrictions were lifted [18]. Despite these global insights, data from Africa remains sparse. In Africa, the RSV burden is likely underestimated due to limited diagnostic capacity.

This systematic review aims to describe the epidemiology of RSV infection in Africa during and after the Covid‐19 pandemic. By synthesizing available data, we assess the impact of pandemic‐related public health measures on RSV transmission and compare these findings with global trends. Our results aim to inform future strategies for RSV prevention, control, and surveillance on the continent.

## 2 Methods

### 2.1 Search Strategy

This systematic review was conducted following the PRISMA (Preferred Reporting Items for Systematic Reviews and Meta‐Analyses) guidelines (Table S1) [19]. The protocol was registered in the PROSPERO database under the number CRD42023486679. We developed search strategies for Medline (Ovid), Embase (Ovid), Global Health (Ovid), Web of Science, and Africa Index Medicus, targeting articles published from the inception of these databases to March 5, 2024 (Table S2). The search terms included “respiratory syncytial virus,” and “Africa”. Publications in both English and French were considered. A manual search was conducted in the bibliographies of the articles included in this review to ensure comprehensive coverage of the literature.

### 2.2 Study Selection

This review included studies reporting epidemiological frequency measures of RSV, such as incidence (new RSV cases in a specific population over a given period) and prevalence (proportions of RSV‐positive cases at a specific time). There were no restrictions on the type of study. The review considered data on acute respiratory tract infections from both hospitalized individuals and those not hospitalized, encompassing all populations, whether they sought medical consultation for acute respiratory infections or not. Studies were excluded if they did not present data from Africa, were duplicates, or were reviews, letters, or editorials. We defined precise criteria based on the study's relevance to the COVID‐19 pandemic period. The timeframe considered for the research covers periods from the start of Covid‐19 pandemic to the beginning of 2024. However, studies that began before this period and covered the Covid‐19 wave were also included. In this way, three periods were defined for the purposes of comparison: the pre‐pandemic period (before December 2019), the pandemic period (December 2019–December 2022) and the post‐pandemic period (after December 2022). Identified studies were exported and duplicates removed using EndNote X9. This library was then exported to a RAYYAN account created for this purpose, which facilitated the exclusion of any remaining duplicates and the automatic selection of eligible studies. Two reviewers (M.I. and A.G.) independently screened titles and abstracts to identify potentially relevant articles based on predefined eligibility criteria. Studies that appeared relevant at this stage were further subjected to full‐text reviews independently conducted by the same reviewers. Reasons for exclusion at the full‐text stage were systematically documented. Any discrepancies between reviewers regarding the inclusion of studies were resolved through discussion to reach a consensus.

### 2.3 Data Extraction and Risk of Bias Evaluation

Data extraction was conducted by two independent reviewers (M.I. and A.G.). The data extracted included the author's name, publication year, study design, setting, location type, countries involved, study period, age range, clinical definition of the conditions studied, RSV diagnostic methods used, and sample types. Key characteristics such as sample size and outcomes related to RSV infection (e.g., incidence and prevalence) were also gathered. To assess the risk of bias in the included studies, we used the Hoy et al. checklist for prevalence studies [20]. This tool evaluates several critical aspects of study, including the representativeness of the study population, the adequacy of inclusion criteria, and the reliability and validity of the RSV detection methods: It is a tool consisting of 10 items or questions used to assess the quality of the studies selected. Items 1–4 assess external quality; items 5–10 assess internal quality and item 10 assesses the quality of data analysis. These items are: (i) Was the study's target population a close representation of the national population in relation to relevant variables? (ii) Was the sampling frame a true or close representation of the target population? (iii) Was some form of random selection used to select the sample, OR was a census undertaken? (iv) Was the likelihood of nonresponse bias minimal? Internal validity (v) Were data collected directly from the subjects (as opposed to a proxy)? (vi) Was an acceptable case definition used in the study? (vii) Was the study instrument that measured the parameter of interest shown to have validity and reliability? (viii) Was the same mode of data collection used for all subjects? (ix). Was the length of the shortest prevalence period for the parameter of interest appropriate? (x) Were the numerator(s) and denominator(s) for the parameter of interest appropriate? Each criterion was scored, and a total risk of bias score was calculated for each study. The final risk of bias was categorized as low (7–10), moderate (4–6), or high (0–3) based on the total score (Table S5). Discrepancies between reviewers were resolved through consensus.

Our meta‐analytical approach focused on assessing the prevalence and incidence of RSV by extracting data from the included studies. This involved calculating pooled estimates under a random effects model due to the high heterogeneity observed among the study results.

### 2.4 Data Analysis [21]

Data were comprehensively analyzed and presented using forest plots. We conducted meta‐analyses on the prevalence and incidence of RSV, expecting high heterogeneity among the included studies. To manage this, random effects (RE) models with double arcsine transformation were employed to stabilize variances, and pooled estimates were presented as proportions with corresponding confidence intervals (CI) [22]. Heterogeneity across studies was quantified using *I*² statistics [23]. Publication bias was evaluated through Egger's test [24]. We further explored sources of heterogeneity through subgroup analyses based on various categories such as setting (community‐based vs. hospital‐based), hospital details (inpatients vs. outpatients), location details (rural vs. urban), and geographical and economic stratifications (countries, United Nations (UN) regions, World Bank income groups). Analyses were stratified by pandemic era (pre‐pandemic, during pandemic, post‐pandemic), age range (all ages vs. children), and clinical definitions. All analyses were performed using the statistical software R version 4.0.3 (2020‐10‐10).

## 3 Results

### 3.1 Review Process

**Figure 1:**
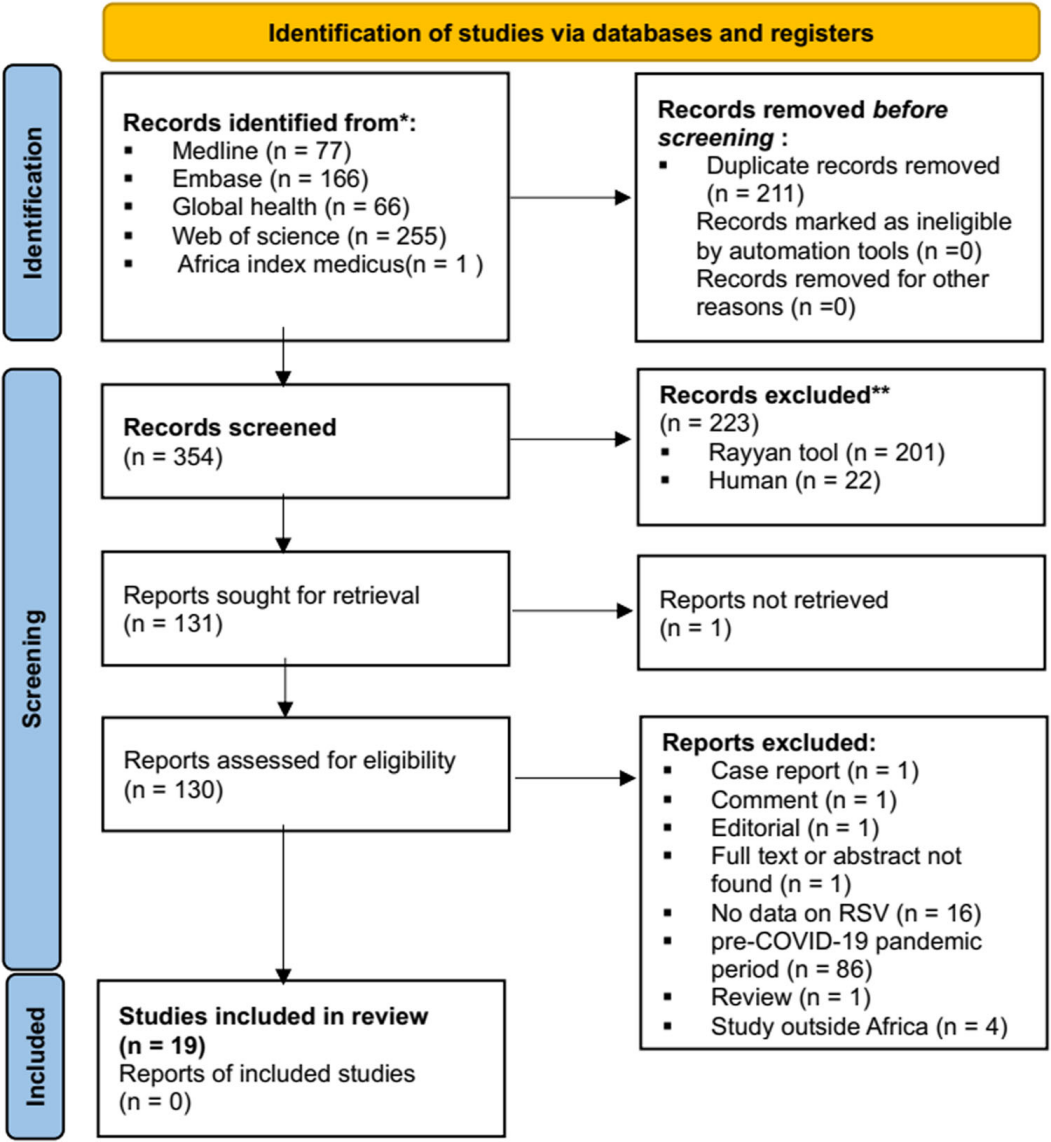
PRISMA 2020 flow diagram for literature search and selection.

Our systematic review was conducted by searching several databases. We searched in Medline (Ovid), Embase (Ovid), Global Health (Ovid), Web of Science, and Africa Index Medicus, yielding a total of 565 records. After removing 211 duplicates, 354 records were screened, and 133 full‐text articles were assessed for eligibility (Figure 1). Of these, 19 studies met the inclusion criteria for our review. The most common reasons for exclusion included data from the pre‐COVID‐19 pandemic period (88 studies excluded), absence of specific data on RSV (*n* = 16), and publications categorized as case reports, comments, editorials, or reviews (*n* = 4).

### 3.2 Characteristics of Included Studies

The included studies encompassed different research designs, with the majority being cross‐sectional (78.95%), followed by cohort (15.79%), and a single case‐control study (5.26%) (Table S3). Sample collection was predominantly prospective (89.47%). The settings of these studies were mainly hospital‐based (84.21%), with a smaller proportion conducted in community settings (15.79%). Among the hospital‐based studies, 47.37% involved only inpatients, while 21.05% were limited to outpatients, and 15.79% covered both inpatients and outpatients. The studies were distributed across five UN regions, with representation as follows: Southern Africa (26.32%), Northern Africa (26.32%), Eastern Africa (31.58%), Western Africa (10.53%), and Middle Africa (5.26%). Studies were also distributed among low‐income (31.58%), lower‐middle‐income (36.84%), and upper‐middle‐income countries (31.58%). All studies spanned the years 2013 to 2023, with their distribution across the pandemic timeline showing 42.11% during the pandemic, 15.79% post‐pandemic, and another 42.11% covering both pre‐pandemic and pandemic periods. The studies focused on all age groups, with a slight majority on all ages (47.37%) and the remainder specifically on children (42.11%). All studies used real‐time RT‐PCR to identify RSV. Sample types varied, with nasopharyngeal swabs being the most common method of sample collection. The risk of bias was predominantly low across the studies (84.21%) (Table S4).

### 3.3 RSV Prevalence Meta‐Analysis in Africa

**Figure 2:**
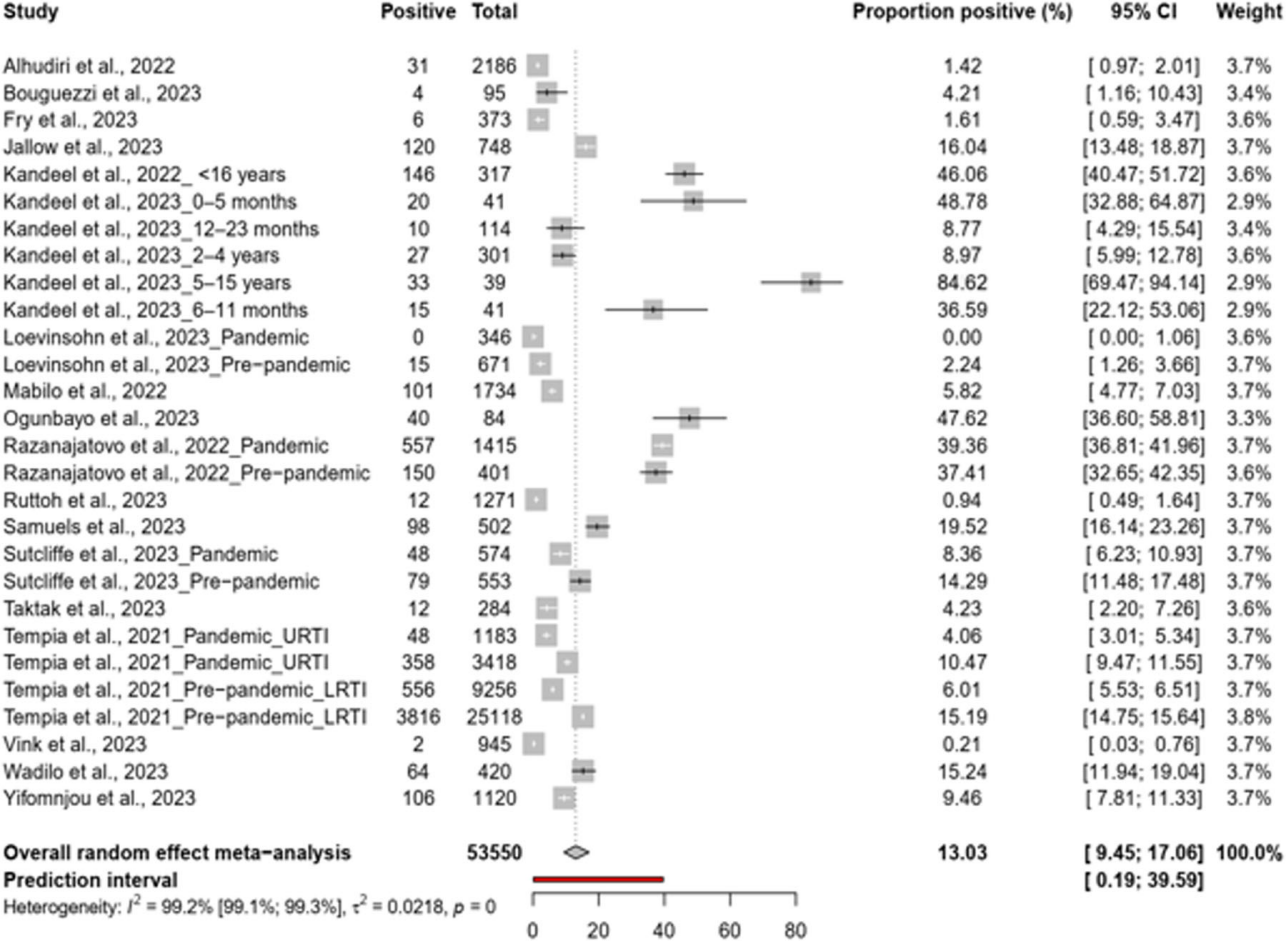
RSV prevalence in Africa before and during the COVID‐19 pandemic.

We ran analysis from 28 data points involving 53,550 cases (Figure 2). The pooled prevalence of RSV was 13% with a 95% confidence interval (CI) ranging from 9.5% to 17.1%. The analysis revealed high heterogeneity among the studies (*I*² = 99.2% [95% CI 99.1–99.3]). The Egger's test for publication bias resulted in a *p* value of 0.745, suggesting no significant evidence of publication bias in the reporting of RSV prevalence data.

To elucidate these potential sources of discrepancy, a subgroup analysis was performed. The results of the subgroup analysis are shown in Table 1.

**Table 1:** Subgroup analyses of the prevalence of RSV in Africa before and during the COVID‐19 pandemic.

	Prevalence. % (95% CI)	*N* studies	*N* participants	*p* value difference subtypes
Setting				< 0.001*
Community‐based	0.9 [0.2–2.2]	3	3504	
Hospital‐based	15.3 [11.5–19.5]	25	50,046	
Hospital details				0.001*
Inpatients	21.3 [15.4–27.9]	11	34,252	
Outpatients	11.3 [7.1–16.2]	12	14,390	
Location details				0.20
Rural	11.1 [6–17.6]	2	1127	
Urban	17.3 [10–25.9]	13	5013	
Countries				< 0.001*
Egypt	36.6 [15.5–60.7]	6	853	
Madagascar	38.9 [36.7–41.2]	2	1816	
South Africa	9.8 [5.7–14.8]	7	41,166	
Tunisia	4.1 [2.3–6.5]	2	379	
Zambia	4.5 [0.4–12.4]	4	2144	
UN regions				0.04*
Eastern Africa	9 [1.6–21.4]	9	6596	
Northern Africa	22 [8.2–40]	9	3418	
Southern Africa	9.8 [5.7–14.8]	7	41,166	
Western Africa	17.6 [14.3–21.1]	2	1250	
World bank income groups				0.11
Low‐income countries	11.4 [2.9–24.4]	9	5827	
Lower‐middle‐income countries	19.6 [10.1–31.2]	11	4371	
Upper‐middle‐income countries	8.4 [4.5–13.4]	8	43,352	
Era				0.07
Pandemic	8.8 [3.7–15.7]	13	14,076	
Post‐pandemic	30.6 [12.4–52.5]	7	948	
Pre‐pandemic	12.9 [6.8–20.6]	5	35,999	
Age range				< 0.001*
All ages	5.9 [3.3–9.1]	14	48,462	
Children	29.8 [18.8–42.1]	12	4048	
Clinical definition				0.49
LRTI	13.4 [8.4–19.4]	6	30,069	
URTI	10.8 [6.9–15.5]	14	14,768	

Abbreviations: LRTI, lower respiratory tract infections; URTI, upper respiratory tract infections.

* *p* value significant.

We found that the age subgroups of the study population (children–adults), the study setting (community or hospital), the patient category (inpatient or outpatient), the study country and the UN subgroups all had an influence on the observed heterogeneity.

The prevalence of RSV was higher in hospital settings (15.3% [95% CI: 11.5–19.5]) compared to community settings (0.9% [95% CI: 0.2–2.2]), with inpatients showing a higher rate (21.3% [95% CI: 15.4–27.9]) than outpatients (11.3% [95% CI: 7.1–16.2]) (Table 1). There were differences among countries and UN regions, with the highest rates observed in Madagascar (38.9% [95% CI: 36.7–41.2]) and Egypt (36.6% [95% CI: 15.5–60.7]). During the pandemic, prevalence was lower (8.8% [95% CI: 3.7–15.7]) compared to the post‐pandemic period (30.6% [95% CI: 12.4–52.5]). Children experienced a higher prevalence (29.8% [95% CI: 18.8–42.1]) than all ages combined. The type of clinical setting, low respiratory tract infections versus upper respiratory tract infections (LRTI vs. URTI), showed less pronounced differences.

The remaining subgroups, that is, location (rural or urban), period (pre‐pandemic, pandemic and post‐pandemic), World Bank income group and case definition, also appear to play a role in the difference observed. However, this difference is not statistically significant (*p* value 0.1–0.49).

### 3.4 Proportion of RSV Antigenic Subgroups

Two studies reported the proportion of RSV antigenic subgroups. Of 183 strains of RSV identified, 132 (72.13%) were RSV B compared with 51 (27.83%) RSV A and one undetermined case.

### 3.5 RSV Incidence Meta‐Analysis in Africa

**Figure 3:**
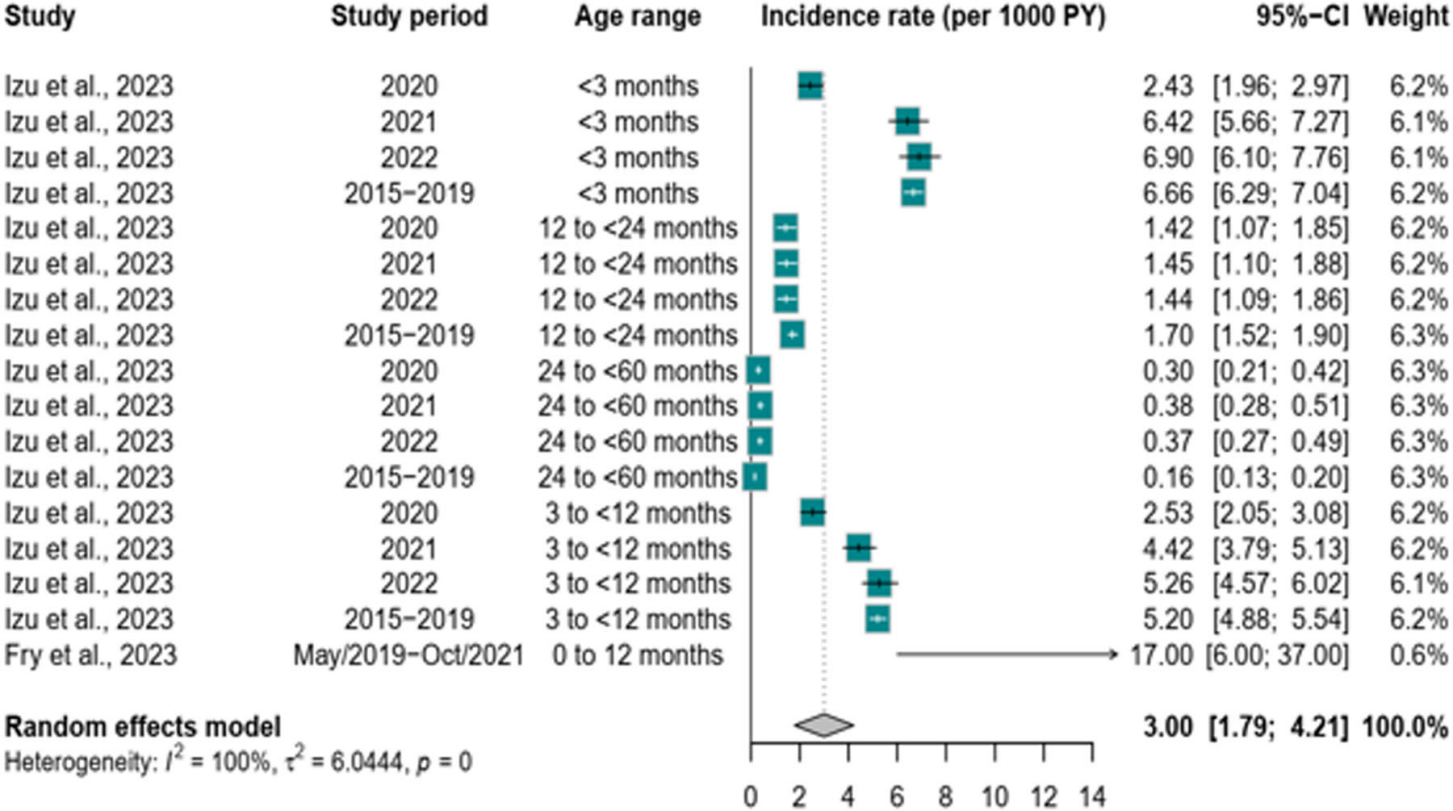
RSV incidence in Africa before and during the COVID‐19 pandemic.

The incidence per 1000 person‐years was from two studies providing 17 data points (Figure 3). The study by Izu et al. [25], provides a detailed breakdown of incidences ranging according to period before, during, and after the COVID‐19 pandemic from 0.16 [95% CI: 0.13; 0.20] per 1000 person‐years to 6.90 [95% CI: 6.10; 7.76] per 1000 person‐years. The pooled analysis under a random effects model calculated an overall incidence rate of 3.00 [95% CI: 1.79; 4.20] per 1000 person‐years. The high *I* ^2^ value of 99.5% [95% CI: 99.4%; 99.6%] indicated substantial heterogeneity.

## 4 Discussion

We conducted this systematic review and meta‐analysis to assess the impact of the Covid‐19 pandemic on RSV epidemiology in Africa. Overall, the estimated prevalence and incidence were 13.03% and 3 per 1000 person‐years, respectively. Prevalence varied significantly by patient age, hospitalization status, and country. RSV infection was notably higher in children than the general population (29.8 vs. 5.9%) and in hospitalized compared to nonhospitalized patients (15.3% vs. 0.9%).

The estimated prevalence in our study was 13.03%, which aligns with pre‐pandemic data from Africa, where Kenmoe et al. reported a prevalence of 14.6% among all age groups with acute respiratory infections [26]. Higher rates were observed in children (18.5%), which may explain the elevated prevalence reported in other regions focused on pediatric populations. In the Middle East and North Africa (MENA) region, Yassine et al. found a prevalence of 24.4% in children, with peaks over 60% in some countries [27]. In Europe, Suleiman‐Martos et al. reported an even higher pooled prevalence of 46% among children under five [28]. Despite the use of similar diagnostic methods (RT‐PCR), these variations likely reflect differences in age groups studied, clinical severity, climate, and environmental risk factors.

Subgroup analysis confirmed age as a key determinant. Fourteen studies reporting prevalence across all ages yielded a pooled estimate of 5.9%, whereas prevalence in children alone was substantially higher (29.8%). This age disparity aligns with known RSV epidemiology, which shows increased susceptibility among young children, particularly those under 2 years [3, 29].

RSV infections were more frequently reported in hospital settings than in community settings (15.3% [95% CI 11.5–19.5] vs. 0.9% [0.2–2.2]; *p* < 0.001). Among hospital‐based populations, the prevalence was higher in inpatients than in outpatients (21.3% [15.4–27.9] vs. 11.3% [7.1–16.2]; *p* = 0.012). Several factors may explain this difference. First, hospital‐based studies benefit from greater testing availability and clinical prioritization, especially during the COVID‐19 pandemic when testing in the community was limited. Second, patients admitted to the hospital are generally more vulnerable to severe forms of RSV infection, which increases the likelihood of detection [30]. Third, nosocomial transmission may have contributed to higher hospital prevalence, as RSV can spread via contaminated surfaces or through infected healthcare workers [31]. These findings are consistent with previous large‐scale studies, such as that by Staadegaard and colleagues, which reported higher RSV prevalence among hospitalized patients across 15 countries [32]. On the other hand, Xie et al. found no difference in the prevalence of RSV infection in the two groups (hospitalized and nonhospitalized) [33].

The Covid‐19 pandemic profoundly disrupted RSV transmission dynamics. In our review, RSV prevalence fell from 12.9% pre‐pandemic to 8.8% during the pandemic, before rising sharply to 30.6% post‐pandemic. This pattern reflects global trends. Cong et al. found that RSV‐associated hospitalizations dropped by up to 80% in high‐income countries in 2020, with a return to pre‐pandemic levels in 2021–2022 [17]. Similarly, Leija‐Martínez et al. observed a drastic reduction in RSV prevalence during the lockdown period (5.0%) compared to pre‐pandemic levels (25.6%), followed by a significant post‐pandemic resurgence (42.0%) [34]. Several factors likely contributed to these fluctuations. Non‐pharmaceutical interventions (NPIs) such as school closures, social distancing, mask use, and travel restrictions effectively suppressed RSV circulation [16]. As these measures were relaxed, populations, particularly infants, became more susceptible due to a lack of natural exposure. This “immunity gap” may explain the pronounced post‐pandemic rebound. Despite these variations, our time‐period‐based subgroup analysis did not find statistically significant differences (*p* = 0.071), although the trends remain epidemiologically relevant. This may be due to the limited number of studies reporting prevalence per period and the high heterogeneity among them.

Only two studies reported RSV incidence, with considerable variation (*I*² = 99.5%). Izu et al. reported rates ranging from 0.16 to 6.9 per 1000 person‐years, depending on age, while Fry et al. found an incidence of 17 per 1000 person‐years in infants followed from birth to 1 year during peak transmission seasons. These differences underscore the need for more standardized incidence rate reporting in future studies. [25] Africa bears a disproportionate burden of RSV‐related morbidity and mortality. The continent accounts for more than 4 million RSV infections annually among children under five, and nearly half of global RSV‐related deaths. Most of these deaths occur outside hospital settings due to limited access to healthcare, highlighting the urgent need to strengthen community‐level surveillance and care [4, 35]. Older adults also face a substantial RSV burden. Although underreported, recent global estimates show that RSV‐related deaths and disability‐adjusted life years (DALYs) are increasing in older adults, with mortality rates exceeding those in children under five. Improved data on RSV epidemiology in the elderly is essential to guide public health responses [36, 37]. Effective interventions exist but remain out of reach for many in Africa. Palivizumab reduces severe RSV‐related complications in high‐risk infants, while ribavirin has shown benefit in shortening hospital stays and reducing mortality [38, 39]. However, high costs limit their use in low‐resource settings. Recent real‐world data support the high effectiveness and safety of newer preventive tools. Several long‐acting monoclonal antibodies have demonstrated over 80% effectiveness against RSV‐related emergency visits and hospitalizations in infants, with a strong safety profile [40]. Similarly, RSV vaccines for older adults have shown nearly 80% effectiveness against hospital admissions, with very low incidence of serious adverse events. Although evidence on the RSV maternal vaccine remains limited, no severe safety concerns have been reported to date. These findings offer promising prospects for reducing RSV burden across age groups; however, equitable access and implementation strategies in resource‐limited settings remain critical challenges.

### 4.1 Strengths and Limitations

This review has several strengths. All included studies used RT‐PCR, and most were prospective and rated at low risk of bias. Subgroup analyses allowed us to explore important sources of heterogeneity, including age, hospitalization status, and study setting.

However, limitations remain. Data were drawn from only 12 African countries, limiting generalizability. Substantial heterogeneity across studies (in design, populations, and time periods) may affect the precision of pooled estimates. Additionally, few studies reported incidence data or stratified results by pandemic period, limiting temporal comparisons.

## 5 Conclusion

In summary, this systematic review and meta‐analysis provide valuable evidence on the impact of the COVID‐19 pandemic on RSV prevalence. The resurgence of RSV following the relaxation of COVID‐19 restrictions has been highlighted in several places globally. This highlights the need for ongoing surveillance and public health interventions to mitigate the burden of RSV‐related respiratory infections in the post‐pandemic era.

The high prevalence of RSV observed is more important in the pediatric population in Africa, as has been highlighted by subsequent data. Consequently, prevention and control of RSV infections in children merit greater attention from healthcare providers, researchers, and policy‐makers for effective detection, management and control. Effective interventions are needed to reduce this burden of RSV infection; this warrants in‐depth studies on the burden of hospitalization, aspects of infection at community level, and interventions in older people.

Control interventions should focus on the availability of control agents such as vaccines and monoclonal antibodies.

## Author Contributions

**Moussa Issa:** conceptualization, data curation, formal analysis, supervision, writing – review and editing, writing – original draft, project administration, validation, and methodology. **Adamou Lagare:** conceptualization, methodology, data curation, validation, formal analysis, supervision, project administration, writing – original draft, writing – review and editing. **Goni A. M. Bachir:** data curation, formal analysis, and investigation. **Fatimata Hassane:** investigation and methodology. **Larwanou Harouna Magagi:** investigation and methodology. **Doutchi Mahamadou:** conceptualization and validation. **Haoua Seini:** conceptualization and validation. **Eric Adehossi:** conceptualization, validation, project administration, writing – review and editing, and supervision. **Alzouma Mayaki Zoubeirou:** conceptualization, validation, supervision, writing – review and editing, and project administration.

## Conflicts of Interest

The authors declare no conflicts of interest.

## Transparency Statement

The lead author, Moussa Issa, affirms that this manuscript is an honest, accurate, and transparent account of the study being reported; that no important aspects of the study have been omitted; and that any discrepancies from the study as planned (and, if relevant, registered) have been explained.

## Supporting information


**Supporting Table 1:** Preferred reporting items for systematic reviews and meta‐analyses checklist. **Supporting Table 2:** Search strategy. **Supporting Table 3:** Items for risk of bias assessment. **Supporting Table 4:** Individual characteristics of included studies. **Supporting Table 5:** Risk of bias assessment.

## Data Availability

The authors confirm that the data supporting the findings of this study are available within the article and its Supporting Information S1.
