# Supplementary material for: Respiratory Syncytial Virus Epidemiology During and After Covid‐19 Pandemic in Africa: Systematic Review and Meta‐Analysis
Source: Health Sci Rep. 2025 Nov 26;8(12):e71583. doi: 10.1002/hsr2.71583 (PMC12657640; doi:10.1002/hsr2.71583)
Supplement: Supplementary file 1 — Supporting Table 1: Preferred reporting items for systematic reviews and meta‐analyses checklist. Supporting Table 2: Search strategy. Supporting Table 3: Items for risk of bias assessment. Supporting Table 4: Individual characteristics of included studies. Supporting Table 5: Risk of bias assessment. [file HSR2-8-e71583-s001.docx]

Supplemental Material

Table of Contents

[Supplementary Table 1 : Preferred reporting items for systematic reviews and meta-analyses checklist 2](#_Toc176188840)

[Supplementary Table 2: Search strategy 5](#_Toc176188841)

[Supplementary Table 3: Items for risk of bias assessment 8](#_Toc176188842)

[Supplementary Table 4: Individual characteristics of included studies 9](#_Toc176188843)

[Supplementary Table 5: Risk of bias assessment 10](#_Toc176188844)

Supplementary Table 1 : Preferred reporting items for systematic reviews and meta-analyses checklist

| **Section and Topic** | **Item #** | **Checklist item** | **Location where item is reported** |
| --- | --- | --- | --- |
| **TITLE** | | |  |
| Title | 1 | Identify the report as a systematic review. | Page 1 |
| **ABSTRACT** | | |  |
| Abstract | 2 | See the PRISMA 2020 for Abstracts checklist. | Pages 2-3 |
| **INTRODUCTION** | | |  |
| Rationale | 3 | Describe the rationale for the review in the context of existing knowledge. | Pages 4-5 |
| Objectives | 4 | Provide an explicit statement of the objective(s) or question(s) the review addresses. | Page 5 |
| **METHODS** | | |  |
| Eligibility criteria | 5 | Specify the inclusion and exclusion criteria for the review and how studies were grouped for the syntheses. | Page 5 |
| Information sources | 6 | Specify all databases, registers, websites, organisations, reference lists and other sources searched or consulted to identify studies. Specify the date when each source was last searched or consulted. | Page 6 |
| Search strategy | 7 | Present the full search strategies for all databases, registers and websites, including any filters and limits used. | S2 Table |
| Selection process | 8 | Specify the methods used to decide whether a study met the inclusion criteria of the review, including how many reviewers screened each record and each report retrieved, whether they worked independently, and if applicable, details of automation tools used in the process. | Page 6 |
| Data collection process | 9 | Specify the methods used to collect data from reports, including how many reviewers collected data from each report, whether they worked independently, any processes for obtaining or confirming data from study investigators, and if applicable, details of automation tools used in the process. | Page 6 |
| Data items | 10a | List and define all outcomes for which data were sought. Specify whether all results that were compatible with each outcome domain in each study were sought (e.g. for all measures, time points, analyses), and if not, the methods used to decide which results to collect. | Page 6 |
|  | 10b | List and define all other variables for which data were sought (e.g. participant and intervention characteristics, funding sources). Describe any assumptions made about any missing or unclear information. | Page 6 |
| Study risk of bias assessment | 11 | Specify the methods used to assess risk of bias in the included studies, including details of the tool(s) used, how many reviewers assessed each study and whether they worked independently, and if applicable, details of automation tools used in the process. | Page 6 |
| Effect measures | 12 | Specify for each outcome the effect measure(s) (e.g. risk ratio, mean difference) used in the synthesis or presentation of results. | Page 6 |
| Synthesis methods | 13a | Describe the processes used to decide which studies were eligible for each synthesis (e.g. tabulating the study intervention characteristics and comparing against the planned groups for each synthesis (item #5)). | Page 6 |
|  | 13b | Describe any methods required to prepare the data for presentation or synthesis, such as handling of missing summary statistics, or data conversions. | Page 6 |
|  | 13c | Describe any methods used to tabulate or visually display results of individual studies and syntheses. | Page 6 |
|  | 13d | Describe any methods used to synthesize results and provide a rationale for the choice(s). If meta-analysis was performed, describe the model(s), method(s) to identify the presence and extent of statistical heterogeneity, and software package(s) used. | Page 6 |
|  | 13e | Describe any methods used to explore possible causes of heterogeneity among study results (e.g. subgroup analysis, meta-regression). | Note done |
|  | 13f | Describe any sensitivity analyses conducted to assess robustness of the synthesized results. | Note done |
| Reporting bias assessment | 14 | Describe any methods used to assess risk of bias due to missing results in a synthesis (arising from reporting biases). | Page 6 |
| Certainty assessment | 15 | Describe any methods used to assess certainty (or confidence) in the body of evidence for an outcome. | Page 6 |
| **RESULTS** | | |  |
| Study selection | 16a | Describe the results of the search and selection process, from the number of records identified in the search to the number of studies included in the review, ideally using a flow diagram. | Page 6 |
|  | 16b | Cite studies that might appear to meet the inclusion criteria, but which were excluded, and explain why they were excluded. | Page 6 |
| Study characteristics | 17 | Cite each included study and present its characteristics. | Table 1 |
| Risk of bias in studies | 18 | Present assessments of risk of bias for each included study. | S3 Table |
| Results of individual studies | 19 | For all outcomes, present, for each study: (a) summary statistics for each group (where appropriate) and (b) an effect estimate and its precision (e.g. confidence/credible interval), ideally using structured tables or plots. | Page 7-8 |
| Results of syntheses | 20a | For each synthesis, briefly summarise the characteristics and risk of bias among contributing studies. | Page 8 |
|  | 20b | Present results of all statistical syntheses conducted. If meta-analysis was done, present for each the summary estimate and its precision (e.g. confidence/credible interval) and measures of statistical heterogeneity. If comparing groups, describe the direction of the effect. | *Figure 1* |
|  | 20c | Present results of all investigations of possible causes of heterogeneity among study results. | Figure 2 |
|  | 20d | Present results of all sensitivity analyses conducted to assess the robustness of the synthesized results. | Note done |
| Reporting biases | 21 | Present assessments of risk of bias due to missing results (arising from reporting biases) for each synthesis assessed. | S3 table |
| Certainty of evidence | 22 | Present assessments of certainty (or confidence) in the body of evidence for each outcome assessed. | Note done |
| **DISCUSSION** | | |  |
| Discussion | 23a | Provide a general interpretation of the results in the context of other evidence. | Page 9 |
|  | 23b | Discuss any limitations of the evidence included in the review. | Pages10-11 |
|  | 23c | Discuss any limitations of the review processes used. | Page 11 |
|  | 23d | Discuss implications of the results for practice, policy, and future research. | Page 11 |
| **OTHER INFORMATION** | | |  |
| Registration and protocol | 24a | Provide registration information for the review, including register name and registration number, or state that the review was not registered. | Page 5 |
|  | 24b | Indicate where the review protocol can be accessed, or state that a protocol was not prepared. | Page 5 |
|  | 24c | Describe and explain any amendments to information provided at registration or in the protocol. | Page 5 |
| Support | 25 | Describe sources of financial or non-financial support for the review, and the role of the funders or sponsors in the review. | Page 2 |
| Competing interests | 26 | Declare any competing interests of review authors. | Page 13 |
| Availability of data, code and other materials | 27 | Report which of the following are publicly available and where they can be found: template data collection forms; data extracted from included studies; data used for all analyses; analytic code; any other materials used in the review. | Note done |

*From:*  Page MJ, McKenzie JE, Bossuyt PM, Boutron I, Hoffmann TC, Mulrow CD, et al. The PRISMA 2020 statement: an updated guideline for reporting systematic reviews. BMJ 2021;372:n71. doi: 10.1136/bmj.n71

**Supplementary Table 2: Search strategy**

| **Database** |  | Search (done on Mars 05, 2024) | Items |
| --- | --- | --- | --- |
| **Medline (Ovid)** | 1 | exp Respiratory Syncytial Virus/ or exp Respiratory Syncytial Virus Infections/ or exp Respiratory Syncytial Virus, Human/ | 14098 |
|  | 2 | (RSV or Orthopneumovirus).mp. | 15871 |
|  | 3 | ((exp "Africa South of the Sahara"/ or exp "Democratic Republic of the Congo"/ or exp Africa/ or exp Africa South of the Sahara/ or exp Africa, Central/ or exp Africa, Eastern/ or exp Africa, Northern/ or exp Africa, Southern/ or exp Africa, Western/ or exp Algeria/ or exp Angola/ or exp Benin/ or exp Botswana/ or exp Burkina Faso/ or exp Burundi/ or exp Cabo Verde/ or exp Cameroon/ or exp Central African Republic/ or exp Chad/ or exp Comoros/ or exp Congo/ or exp Cote d'Ivoire/ or exp Djibouti/ or exp Egypt/ or exp Equatorial Guinea/ or exp Eritrea/ or exp Eswatini/ or exp Ethiopia/ or exp Gabon/ or exp Gambia/ or exp Ghana/ or exp Guinea/ or exp Guinea-Bissau/ or exp Kenya/ or exp Lesotho/ or exp Liberia/ or exp Libya/ or exp Madagascar/ or exp Malawi/ or exp Mali/ or exp Mauritania/ or exp Mauritius/ or exp Morocco/ or exp Mozambique/ or exp Namibia/ or exp Niger/ or exp Nigeria/ or exp Reunion/ or exp Rwanda/ or exp Sao Tome/) and Principe/) or exp Senegal/ or exp Seychelles/ or exp Sierra Leone/ or exp Somalia/ or exp South Africa/ or exp South Sudan/ or exp Sub-Saharan African People/ or exp Sudan/ or exp Tanzania/ or exp Togo/ or exp Tunisia/ or exp Uganda/ or exp Zambia/ or exp Zimbabwe/ | 117354 |
|  | 4 | ((("Africa South of the Sahara" or "Democratic Republic of the Congo" or Africa or Africa South of the Sahara or Algeria or Angola or Benin or Botswana or Burkina Faso or Burundi or Cabo Verde or Cameroon or Cape Verde or Central Africa or Central African or Central African Republic or Chad or Comoros or Congo or Cote d'Ivoire or Democratic Republic of Congo or Djibouti or East Africa or East African or Eastern Africa or Eastern African or Egypt or Equatorial Guinea or Eritrea or Eswatini or Ethiopia or Gabon or Gambia or Ghana or Guinea or Guinea Bissau or Guinea-Bissau or Ivory Coast or Jamahiriya or Kenya or Lesotho or Liberia or Libya or Madagascar or Malawi or Mali or Mauritania or Mauritius or Mayotte or Morocco or Mozambique or Namibia or Niger or Nigeria or North Africa or North African or Northern Africa or Northern African or Principe or Reunion or Rwanda or Sao Tome or Sao Tome) and Principe) or Senegal or Seychelles or Sierra Leone or Somalia or South Africa or South African or South Sudan or Southern Africa or Southern African or St Helena or Sub-Saharan African People or Sudan or Swaziland or Tanzania or Togo or Tunisia or Uganda or West Africa or West African or Western Africa or Western African or Western Sahara or Zaire or Zambia or Zimbabwe).mp. | 188282 |
|  | 5 | 1 or 2 | 21180 |
|  | 6 | 3 or 4 | 188294 |
|  | 7 | 5 and 6 | 173 |
|  | 8 | limit 7 to yr="2019 -Current" | 77 |
|  |  |  |  |
| **Embase (Ovid)** | 1 | exp human respiratory syncytial virus/ | 9597 |
|  | 2 | (respiratory syncytial virus or rsv or orthopneumovirus).mp. | 34815 |
|  | 3 | ((exp Africa/ or exp Africa south of the Sahara/ or exp Algeria/ or exp Angola/ or exp Benin/ or exp Botswana/ or exp Burkina Faso/ or exp Burundi/ or exp Cameroon/ or exp Cape Verde/ or exp Central Africa/ or exp Central African Republic/ or exp Chad/ or exp Comoros/ or exp Congo/ or exp Cote d'Ivoire/ or exp Democratic Republic Congo/ or exp Djibouti/ or exp Egypt/ or exp Equatorial Guinea/ or exp Eritrea/ or exp Eswatini/ or exp Ethiopia/ or exp Gabon/ or exp Gambia/ or exp Ghana/ or exp Guinea/ or exp Guinea-Bissau/ or exp Kenya/ or exp Lesotho/ or exp Liberia/ or exp Libyan Arab Jamahiriya/ or exp Madagascar/ or exp Malawi/ or exp Mali/ or exp Mauritania/ or exp Mauritius/ or exp Mayotte/ or exp Morocco/ or exp Mozambique/ or exp Namibia/ or exp Niger/ or exp Nigeria/ or exp North Africa/ or exp Reunion/ or exp Rwanda/ or exp Saint Helena/ or exp Sao Tome/) and Principe/) or exp Senegal/ or exp Seychelles/ or exp Sierra Leone/ or exp Somalia/ or exp South Africa/ or exp South Sudan/ or exp sub-Saharan African/ or exp Sudan/ or exp Swaziland/ or exp Tanzania/ or exp Togo/ or exp Tunisia/ or exp Uganda/ or exp Western Sahara/ or exp Zaire/ or exp Zambia/ or exp Zimbabwe/ | 176822 |
|  | 4 | ((("Africa South of the Sahara" or "Democratic Republic of the Congo" or Africa or Africa South of the Sahara or Algeria or Angola or Benin or Botswana or Burkina Faso or Burundi or Cabo Verde or Cameroon or Cape Verde or Central Africa or Central African or Central African Republic or Chad or Comoros or Congo or Cote d'Ivoire or Democratic Republic Congo or Democratic Republic of Congo or Djibouti or East Africa or East African or Eastern Africa or Eastern African or Egypt or Equatorial Guinea or Eritrea or Eswatini or Ethiopia or Gabon or Gambia or Ghana or Guinea or Guinea Bissau or Guinea-Bissau or Ivory Coast or Jamahiriya or Kenya or Lesotho or Liberia or Libya or Libyan Arab Jamahiriya or Madagascar or Malawi or Mali or Mauritania or Mauritius or Mayotte or Morocco or Mozambique or Namibia or Niger or Nigeria or North Africa or North African or Northern Africa or Northern African or Principe or Reunion or Rwanda or Saint Helena or Sao Tome or Sao Tome) and Principe) or Senegal or Seychelles or Sierra Leone or Somalia or South Africa or South African or South Sudan or Southern Africa or Southern African or St Helena or sub-Saharan African or Sub-Saharan African People or Sudan or Swaziland or Tanzania or Togo or Tunisia or Uganda or West Africa or West African or Western Africa or Western African or Western Sahara or Zaire or Zambia or Zimbabwe).mp. | 229768 |
|  | 5 | 1 or 2 | 34815 |
|  | 6 | 3 or 4 | 243400 |
|  | 7 | 5 and 6 | 334 |
|  | 8 | limit 7 to yr="2019 -Current" | 166 |
|  |  |  |  |
| **Global Health (Ovid)** | 1 | (Respiratory Syncytial Virus Infections or RSV or Orthopneumovirus).mp. | 6662 |
|  | 2 | ((("Africa South of the Sahara" or "Democratic Republic of the Congo" or Africa or Africa South of the Sahara or Algeria or Angola or Benin or Botswana or Burkina Faso or Burundi or Cabo Verde or Cameroon or Cape Verde or Central Africa or Central African or Central African Republic or Chad or Comoros or Congo or Cote d'Ivoire or Democratic Republic Congo or Democratic Republic of Congo or Djibouti or East Africa or East African or Eastern Africa or Eastern African or Egypt or Equatorial Guinea or Eritrea or Eswatini or Ethiopia or Gabon or Gambia or Ghana or Guinea or Guinea Bissau or Guinea-Bissau or Ivory Coast or Jamahiriya or Kenya or Lesotho or Liberia or Libya or Libyan Arab Jamahiriya or Madagascar or Malawi or Mali or Mauritania or Mauritius or Mayotte or Morocco or Mozambique or Namibia or Niger or Nigeria or North Africa or North African or Northern Africa or Northern African or Principe or Reunion or Rwanda or Saint Helena or Sao Tome or Sao Tome) and Principe) or Senegal or Seychelles or Sierra Leone or Somalia or South Africa or South African or South Sudan or Southern Africa or Southern African or St Helena or sub-Saharan African or Sub-Saharan African People or Sudan or Swaziland or Tanzania or Togo or Tunisia or Uganda or West Africa or West African or Western Africa or Western African or Western Sahara or Zaire or Zambia or Zimbabwe).mp. | 183281 |
|  | 3 | 1 and 2 | 174 |
|  | 4 | limit 3 to yr="2019 -Current" | 66 |
|  |  |  |  |
|  |  |  |  |
| **Web of Science** | 1 | Topic = (Respiratory Syncytial Virus OR Respiratory Syncytial Virus Infections OR RSV OR Orthopneumovirus) AND ("Africa South of the Sahara" or "Democratic Republic of the Congo" or Africa or Africa South of the Sahara or Algeria or Angola or Benin or Botswana or Burkina Faso or Burundi or Cabo Verde or Cameroon or Cape Verde or Central Africa or Central African or Central African Republic or Chad or Comoros or Congo or Cote d'Ivoire or Democratic Republic Congo or Democratic Republic of Congo or Djibouti or East Africa or East African or Eastern Africa or Eastern African or Egypt or Equatorial Guinea or Eritrea or Eswatini or Ethiopia or Gabon or Gambia or Ghana or Guinea or Guinea Bissau or Guinea-Bissau or Ivory Coast or Jamahiriya or Kenya or Lesotho or Liberia or Libya or Libyan Arab Jamahiriya or Madagascar or Malawi or Mali or Mauritania or Mauritius or Mayotte or Morocco or Mozambique or Namibia or Niger or Nigeria or North Africa or North African or Northern Africa or Northern African or Principe or Reunion or Rwanda or Saint Helena or Sao Tome or Sao Tome and Principe or Senegal or Seychelles or Sierra Leone or Somalia or South Africa or South African or South Sudan or Southern Africa or Southern African or St Helena or sub Saharan Africa or sub Saharan African or sub-Saharan African or Sub-Saharan African People or Sudan or Swaziland or Tanzania or Togo or Tunisia or Uganda or West Africa or West African or Western Africa or Western African or Western Sahara or Zaire or Zambia or Zimbabwe) | 810 |
|  | 2 | limit 1 to yr="2019 -Current" | 255 |
|  |  |  |  |
| Africa Index Medicus | 1 | Respiratory Syncytial Virus OR RSV | 1 |
|  |  |  |  |
|  |  |  |  |
| **Total** |  |  | 565 |
| **Duplicates** |  |  | 211 |
| **Screened** |  |  | 354 |
| **Full text assessment** |  |  | 133 |
| **Included** |  |  | 19 |

Supplementary Table 3: Items for risk of bias assessment

| Hoy et al. tool for cross sectional studies | Yes (1)/No (0) |
| --- | --- |
| 1. Was the study’s target population a close representation of the national population in relation to RSV prevalence? | 1 |
| 2. Was the sampling frame a true or close representation of the target population? | 1 |
| 3. Was some form of random selection used to select the sample, OR was a census undertaken? | 1 |
| 4. Were data collected directly from the subjects (as opposed to a proxy)? |  |
| 5. Was an acceptable inclusion criteria definition used in the study? |  |
| 6. Did the author calculate and respect the expected sample size? |  |
| 7. Was the RSV detection assay shown to have reliability and validity? | 1 |
| 8. Was the same mode of data collection used for all subjects? | 1 |
| 9. Was the length of the study period > or = 1 year? | 1 |
| 10. Were the numerator(s) and denominator(s) for the RSV data appropriate? | 1 |
| Total score | 10 |
| Interpretation of the risk of bias tool   - 7-10: Low risk of bias - 4-6: Moderate risk of bias - 0-3: High risk of bias |  |

Modified from: Hoy D, Brooks P, Woolf A, Blyth F, March L, Bain C, et al. Assessing risk of bias in prevalence studies: modification of an existing tool and evidence of interrater agreement. J Clin Epidemiol. 2012;65: 934–939. doi:10.1016/j.jclinepi.2011.11.014

**Supplementary Table 4: Individual characteristics of included studies**

| **Authors** | **Study design** | **Setting** | **Location type** | **Countries** | **Study period** | **Age range** | **Clinical definition** | **RSV diagnostic method** | **Sample types** |
| --- | --- | --- | --- | --- | --- | --- | --- | --- | --- |
| Alhudiri et al., 2022 | Cross-sectional; Prospetively | Community-based; | Mixed | Libya | December 2021-January 2022. | All ages | Pneumonia, upper respiratory tract infection | Real-time RT-PCR | Nasopharyngeal swabs |
| Bouguezzi et al., 2023 | Cross-sectional; Prospetively | Hospital-based; Inpatients | Urban | Tunisia | October 2022 -January 2023 | > 16 years | SARI | Real-time RT-PCR | Nasopharyngeal swabs or endotracheal aspiration |
| Fry et al., 2023 | Cohort; Prospetively | Community-based; | Urban | South Africa | May 2019- October 2021 | 0-1 year | LRTI | Real-time RT-PCR | Nasal swab |
| Izu et al., 2023 | Cohort; Retroprospectively | Hospital-based; Inpatients | Urban | South Africa | 2015-2022 | <60 months | LRTI | Real-time RT-PCR | Nasopharyngeal swabs or endotracheal aspiration |
| Jallow et al., 2023 | Cross-sectional; Prospetively | Hospital-based; Inpatients | Urban | Senegal | January/2022- December/2022 | 1 month to 31 years | SARI | Real-time RT-PCR | Nasopharyngeal and/or oropharyngeal swabs |
| Kandeel et al., 2022 | Cross-sectional; Prospetively | Hospital-based; Inpatients | Mixed | Egypt | 11:2022 | <16 years | SARI | Real-time RT-PCR | Nasopharyngeal and/or oropharyngeal swabs |
| Kandeel et al., 2023 | Cross-sectional; Prospetively | Hospital-based; Outpatients | Urban | Egypt | 10: 2022 | 0–15 years | ILI | Real-time RT-PCR | Nasopharyngeal and/or oropharyngeal swabs |
| Loevinsohn et al., 2023 | Cross-sectional; Prospetively | Hospital-based; Outpatients | Mixed | Zambia | December/2018-November/2020 | All ages | ILI | Real-time RT-PCR | Nasopharyngeal swabs |
| Mabilo et al., 2022 | Cross-sectional; Prospetively | Hospital-based; Inpatients | Mixed | South Africa | July/2019-December/2020 | All ages | respiratory viral infection | Real-time RT-PCR | Nasopharyngeal swabs, nasal swabs, throat swabs and sputum |
| Ogunbayo et al., 2023 | Cross-sectional; Prospetively | Hospital-based; Inpatients | Urban | South Africa | December/2020-September/2021 | 0-5 years | ARI, SARI | Real-time RT-PCR | Nasopharyngeal and oropharyngeal swabs |
| Razanajatovo et al., 2022 | Cross-sectional; Prospetively | Hospital-based; Inpatients | Mixed | Madagascar | January/2018-May/2022 | 0-5 years | ILI, SARI | Real-time RT-PCR | Nasopharyngeal swabs |
| Ruttoh et al., 2023 | Cross-sectional; Prospetively | Hospital-based; Outpatients | Urban | Kenya | January/2022-December/2022 | All ages | SARSCoV-2 cases | Real-time RT-PCR | Nasopharyngeal swabs |
| Samuels et al., 2023 | Cross-sectional; Prospetively | Hospital-based; Inpatients | Urban | Sierra Leone | October/2020-October/2021 | 0-23 months | ARI | Real-time RT-PCR | Nose and throat swabs |
| Sutcliffe et al., 2023 | Cross-sectional; Prospetively | Hospital-based; Outpatients | Rural | Zambia | 2019-2021 | All ages | ILI | Real-time RT-PCR | Nose and throat swabs |
| Taktak et al., 2023 | Cross-sectional; Retroprospectively | Hospital-based; Inpatients; Outpatients | Urban | Tunisia | October/2020-May/2021 | All ages | ILI | Real-time RT-PCR | Nasopharyngeal swabs |
| Tempia et al., 2021 | Cross-sectional; Prospetively | Hospital-based; Inpatients; Outpatients | Mixed | South Africa | 2013-2020 | All ages | ILI/SARI | Real-time RT-PCR | nasopharyngeal aspirates or nasopharyngeal (NP) and oropharyngeal (OP) swabs |
| Vink et al., 2023 | Cohort; Prospetively | Community-based; | Mixed | Malawi | February/2021-April/2022. | age ≥15 years | ILI | Real-time RT-PCR | Nasal and throat swabs |
| Wadilo et al., 2023 | Case control; Prospetively | Hospital-based; Inpatients | Mixed | Ethiopia | September/2019-May/2022 | 0-5 years | ILI | Real-time RT-PCR | Naso/Oropharyngeal swabs |
| Yifomnjou et al., 2023 | Cross-sectional; Prospetively | Hospital-based; Inpatients; Outpatients | Urban | Cameroon | March/2020-October/2021 | All ages | ILI, SARI | Real-time RT-PCR | nasopharyngeal and/or oropharyngeal swabs |

**Supplementary Table 5: Risk of bias assessment**

| Authors | **Q1** | **Q2** | **Q3** | **Q4** | **Q5** | **Q6** | **Q7** | **Q8** | **Q9** | **Q10** | **Risk of bias** |
| --- | --- | --- | --- | --- | --- | --- | --- | --- | --- | --- | --- |
| Alhudiri et al., 2022 | No | Yes | No | Yes | Yes | No | Yes | Yes | No | Yes | Moderate risk of bias |
| Bouguezzi et al., 2023 | No | Yes | No | Yes | Yes | No | Yes | Yes | No | Yes | Moderate risk of bias |
| Fry et al., 2023 | Yes | Yes | Yes | No | Yes | Yes | Yes | Yes | Yes | Yes | Low risk of bias |
| Izu et al., 2023 | Yes | Yes | Yes | Yes | Yes | Yes | Yes | Yes | Yes | Yes | Low risk of bias |
| Jallow et al., 2023 | No | Yes | No | Yes | Yes | No | Yes | Yes | Yes | Yes | Low risk of bias |
| Kandeel et al., 2022 | Yes | Yes | Yes | Yes | Yes | Yes | Yes | Yes | No | Yes | Low risk of bias |
| Kandeel et al., 2023 | Yes | Yes | Yes | Yes | Yes | Yes | Yes | Yes | No | Yes | Low risk of bias |
| Loevinsohn et al., 2023 | No | Yes | No | Yes | Yes | No | Yes | Yes | Yes | Yes | Low risk of bias |
| Mabilo et al., 2022 | No | Yes | No | Yes | Yes | No | Yes | Yes | Yes | Yes | Low risk of bias |
| Ogunbayo et al., 2023 | No | Yes | No | Yes | Yes | No | Yes | Yes | Yes | Yes | Low risk of bias |
| Razanajatovo et al., 2022 | Yes | Yes | Yes | Yes | Yes | Yes | Yes | Yes | Yes | Yes | Low risk of bias |
| Ruttoh et al., 2023 | No | Yes | No | Yes | Yes | No | Yes | Yes | Yes | Yes | Low risk of bias |
| Samuels et al., 2023 | No | Yes | No | No | Yes | No | Yes | Yes | Yes | Yes | Moderate risk of bias |
| Sutcliffe et al., 2023 | No | Yes | No | Yes | Yes | No | Yes | Yes | Yes | Yes | Low risk of bias |
| Taktak et al., 2023 | No | Yes | No | Yes | Yes | No | Yes | Yes | Yes | Yes | Low risk of bias |
| Tempia et al., 2021 | Yes | Yes | No | Yes | Yes | No | Yes | Yes | Yes | Yes | Low risk of bias |
| Vink et al., 2023 | Yes | Yes | Yes | Yes | Yes | Yes | Yes | Yes | Yes | Yes | Low risk of bias |
| Wadilo et al., 2023 | No | Yes | No | Yes | Yes | No | Yes | Yes | Yes | Yes | Low risk of bias |
| Yifomnjou et al., 2023 | Yes | Yes | Yes | Yes | Yes | No | Yes | Yes | Yes | Yes | Low risk of bias |

Q1: Was the study’s target population a close representation of the national population in relation to RSV prevalence?

Q2: Was the sampling frame a true or close representation of the target population?

Q3: Was some form of random selection used to select the sample, OR was acensus undertaken?

Q4: Were data collected directly from the subjects (as opposed to a proxy)?

Q5: Was an acceptable inclusion criteria definition used in the study?

Q6: Did the author calculate and respect the expected sample size?

Q7: Was the RSV detection assay shown to have reliability and validity?

Q8: Was the same mode of data collection used for all subjects?

Q9: Was the length of the study period > or = 1 year?

Q10: Were the numerator(s) and denominator(s) for the RSV data appropriate?
